# The role of cement augmentation with percutaneous vertebroplasty and balloon kyphoplasty for the treatment of vertebral compression fractures in multiple myeloma: a consensus statement from the International Myeloma Working Group (IMWG)

**DOI:** 10.1038/s41408-019-0187-7

**Published:** 2019-02-26

**Authors:** Charalampia Kyriakou, Sean Molloy, Frank Vrionis, Ronald Alberico, Leonard Bastian, Jeffrey A. Zonder, Sergio Giralt, Noopur Raje, Robert A. Kyle, David G. D. Roodman, Meletios A. Dimopoulos, S. Vincent Rajkumar, Brian B. G. Durie, Evangelos Terpos

**Affiliations:** 1 University College London and Northwick Park Hospitals, London, UK; 2 Royal National Orthopedic Hospital, Stanmore, UK; 3 Moffitt Cancer Center, University of South Florida, Tampa, FL USA; 4 Roswell Park Cancer Center, Buffalo, NY USA; 5 Klinikum Leverkusen, Leverkusen, Germany; 6 Karmanos Cancer Institute, Detroit, MI USA; 7 Memorial Sloan-Kettering Cancer Center, New York, NY USA; 8 Massachusetts General Hospital, Boston, MA USA; 9 Department of Laboratory Medicine and Pathology, Mayo Clinic, Rochester, MN USA; 10 Indiana University, Indianapolis, IN USA; 11 University of Athens School of Medicine, Athens, Greece; 12 Mayo Clinic, Rochester, MN USA; 13 Cedars-Sinai Samuel Oschin Cancer Center, Los Angeles, CA USA

## Abstract

Multiple myeloma (MM) represents approximately 15% of haematological malignancies and most of the patients present with bone involvement. Focal or diffuse spinal osteolysis may result in significant morbidity by causing painful progressive vertebral compression fractures (VCFs) and deformities. Advances in the systemic treatment of myeloma have achieved high response rates and prolonged the survival significantly. Early diagnosis and management of skeletal events contribute to improving the prognosis and quality of life of MM patients. The management of patients with significant pain due to VCFs in the acute phase is not standardised. While some patients are successfully treated conservatively, and pain relief is achieved within a few weeks, a large percentage has disabling pain and morbidity and hence they are considered for surgical intervention. Balloon kyphoplasty and percutaneous vertebroplasty are minimally invasive procedures which have been shown to relieve pain and restore function. Despite increasing positive evidence for the use of these procedures, the indications, timing, efficacy, safety and their role in the treatment algorithm of myeloma spinal disease are yet to be elucidated. This paper reports an update of the consensus statement from the International Myeloma Working Group on the role of cement augmentation in myeloma patients with VCFs.

## Introduction

Multiple myeloma (MM) is a haematologic malignancy characterised by infiltration of the bone marrow by plasma cells which can be associated with lytic bone disease causing severe bone pain, pathological fractures and neurological compromise including cauda equina/spinal cord compression. Up to 90% of the myeloma patients develop osteolytic lesions during the course of their disease^1–3^ and 70% of patients are affected at some stage by osteolytic/osteopenic disease of the spine^4^. Several skeletal events over a patient’s lifetime result in substantial morbidity, mortality^5^ and increased healthcare costs.

The introduction of new targeted therapeutic agents in combination with stem cell transplantation has led to a remarkable evolution in the management of myeloma over the last 2 decades^6,7^. Patients with myeloma are living much longer because of improved treatment of the primary disease. Haematological medical management aims at improving the survival of these patients and maintaining their quality of life (QoL). It is thus especially important to treat the osteolytic bone disease and vertebral compression fractures (VCFs) of the spine in a timely manner. Historically, haematologists treated the pain associated with VCFs with radiotherapy and strong opioids. However, these treatments have their own side effects and do not stabilise the fracture. In many cases, pain remains disabling and some patients develop progressive deformities. Some of the fractures also fail to heal and may result in significant chronic pain.

Treatment of the spine is directed towards keeping the patient pain free, ambulatory and continent. A secondary aim should be to minimise any progressive kyphotic deformity which can lead to a poor QoL. Vertebrae that are severely compressed can lose more than 50% of their original height^8^. Following a spinal fracture, there is an exponential risk of a subsequent one due to an abnormal sagittal balance that ensues and the additional compressive forces on the anterior aspect of the spine^9–11^. Moreover, the patients adopt a kyphotic posture to minimise pain from their VCFs and to compensate for the weakness of the spinal musculature^12–14^. Patients with multiple fractures have reduced activities of daily living, pulmonary and gastric problems and have increased morbidity and mortality^11,13^. The kyphotic posture significantly reduces lung function^15^ and pulmonary disease is the commonest cause of death in women with VCFs^11,16,17^. Another major consequence of the spinal deformities is the psychological impact to these patients who often suffer from depression, anxiety and low self-esteem, with accompanying loss of QoL^18–20^.

The introduction of minimally invasive procedures such as balloon kyphoplasty (BKP) and percutaneous vertebroplasty (PV) has enabled the vast majority of the treated patients to return to a near normal level of function within a very short period of time with excellent pain relief^21–33^.

In 2008, a consensus statement was published by the International Myeloma Working Group (IMWG) on the role of vertebral augmentation with cement in MM^34^. The aim of this paper is to update the previous recommendations by the IMWG considering new published data.

### Overview of vertebral cement augmentation procedures (VCPs), PV and BKP versus alternate options as therapy for VCFs

The PV and BKP have been extensively used in the treatment of painful osteoporotic and cancer-related VCFs. The value of these modalities in treating osteoporotic VCFs was questioned because of the results from two prospective randomised trials that showed no benefit when compared with a sham procedure in relieving pain^35–40^. Kallmes et al. had a simulated surgical procedure as control group without cement augmentation, and in the study by Buchbinder et al., the patients had a sham procedure which entailed injection of a local anaesthetic into the periosteum over the lamina and pedicle. Some of the criticisms of these two studies, the patients most in need of cement augmentation and therefore potentially the ones with the maximum amount of benefit, were possibly excluded from the studies on the basis that they would not allow themselves to be randomised into one of the two groups. The patients enrolled in the studies may have had facet joint-related pain and not the severe pain that one would usually associate with an acute VCF. This may explain why the patients did not show improvement following VP. It is recognised clinically that the severe pain due to an acute VCF subsides if the fracture starts to heal but patients can experience residual pain related to a resultant deformity. The deformity alters the facet joint mechanics and facet-related pain can ensue. Wilson et al. reported that a third of the patients technically suitable for VP for an osteoporotic fracture, responded beneficially to a facet joint injection alone. In this study, the percentage of the enrolled patients may had facet-related pain rather pain from the original fracture^41,42^. Significant reduction in mortality and morbidity using cement augmentation with BKP or VP versus nonsurgical management was reported in retrospective analyses^25,43^.

However, these studies reported outcomes on osteoporotic and not on cancer patients. In the cancer population, prospective and retrospective analyses reported favourable results in the treatment of painful metastatic cancer and myeloma-related VCF’s with PV and BKP^24,29,44–49^. The prospective randomised controlled trial Cancer Patient Fracture Evaluation (CAFE), provided evidence for the superiority of BKP versus non-surgical management (NSM) of painful VCFs. 134 cancer patients were enrolled, of whom 49 had MM. Of these, 22 were randomly assigned to BKP and 27 to NSM^33^. BKP was found to be significantly more favourable than NSM offering rapid and sustained pain relief at 1 year, as well as improved back function, QoL, activity, reduced use of analgesics and bed rest days. Similarly, other studies have reported beneficial results of BKP and PV in rapid pain control, functional and QoL in MM patients with VCFs^24,29–31,45,48,50–52^.

Additional kyphoplasty was more effective than additional radiation or systemic therapy in terms of pain relief, reduction of pain associated disability and of fracture incidence of the entire thoracolumbar spine^44^. It is very important that we do not deny treatment in MM patients that may be very effective in relieving the pain from their acute VCF. They may require VCP (VP or BKP) to give them rapid relief of their pain and return them to function as soon as possible. The pain relief from cement augmentation has been sustained over long post-operative periods in patients with MM^21,30,50,53,54^. Many patients with myeloma who get over the acute fracture pain may benefit from facet joint injections for facet-related pain due to the kyphotic deformity.

The evidence on bracing in the management of osteoporotic VCFs is conflicting and the role of the use of external supportive devices including rigid thoracolumbar spinal orthosis (TLSO) or hyperextension braces is yet to be defined^55^. Splinting of fractures and thermoplastic bracing of spinal deformities has been used for many years to treat disability and pain. Bracing for 8–12 weeks has also been used for the treatment of VCF’s related to myeloma^52^. This may be all that is needed to give the patients pain relief from their acute fracture pain. The thermoplastic brace may also give temporary stability to a fractured spine and to patients with sternal fracture^56^ while chemotherapy is initiated. The most important treatment modality is the systemic anti-myeloma therapy to get the myeloma under control. After one or two cycles of systemic anti-myeloma therapy, cement augmentation (PV and BKP) can be performed if in fact the acute fracture pain is still present. Often these patients have had relief of their acute fracture pain with the thermoplastic brace alone or with the addition of 4–6 weeks of medical/conservative treatment. This means that the patients may not need cement augmentation. Instead if they have chronic pain of a lower intensity over their kyphotic deformity they may benefit from some facet joint injections.

A small group of myeloma patients present with a soft tissue myelomatous mass within the spinal canal that can result in spinal cord or cauda equine compression. These patients often present with neurological deficits and each case needs to be assessed individually. MRI scanning is clearly imperative but a CT scan with soft tissue windows will help to delineate whether the neural compression is due to bone or soft tissue. If the compression is due to a soft tissue mass with associated neurological impairment, then this may be amenable to steroids/chemotherapy and immediate radiotherapy. Patients with cord compression and no neurological deficit may not need radiotherapy because chemotherapy and steroids have been shown to result in excellent resolution of the soft tissue myelomatous mass. Ideally, if patients can be treated with steroids, radiotherapy and chemotherapy and have a very good resolution of their symptoms in 24 h, then they may not need surgical decompression and stabilisation. All decisions regarding patients with spinal cord compression need to be taken into conjunction with an experienced spinal surgeon. Clearly, if a patient has spinal cord or cauda equina compression and has significant neurological sequelae then they may require urgent surgical decompression and associated fixation. The aim however in patients with haematological malignancies should be to try and avoid placement of screws/fixation of the spine. The metalwork has a higher than normal risk of failure because the bone is very weak due to MM causing secondary osteoporosis. In addition, there is a higher risk of metalwork infection because the patients are immunosuppressed during their conventional chemotherapy, immunotherapy and stem cell transplantation. After resolution of the intraspinal mass with chemotherapy, radiotherapy and steroids the fractured vertebra may need to be augmented with cement to treat the acute fracture pain but also to give mechanical support to the anterior and middle columns of the spine^57^ thereby preventing further collapse of the vertebral body. Further collapse, particularly into kyphosis, may lead to spinal cord compromise because of the deformity.

Patients that present with no intraspinal soft tissue mass, but overt bony destruction and dubious spinal stability are another important group of patients. A posterior vertebral wall defect or pedicle/facet fracture may lead one to question the spinal stability in this patient group. Often all that is needed is a spinal brace to keep them out of pain while the spine confers itself stability by producing bridging bony osteophytes^58,59^. This appearance is similar to diffuse idiopathic skeletal hyperostosis^60^. It is a phenomenon that may be accelerated by or the result of treatment with bisphosphonates. This is a very interesting finding and warrants further research to see whether patients with myeloma present a completely different clinical problem than patients with osteolytic metastases due to solid tumours.

**Fig. 1:**
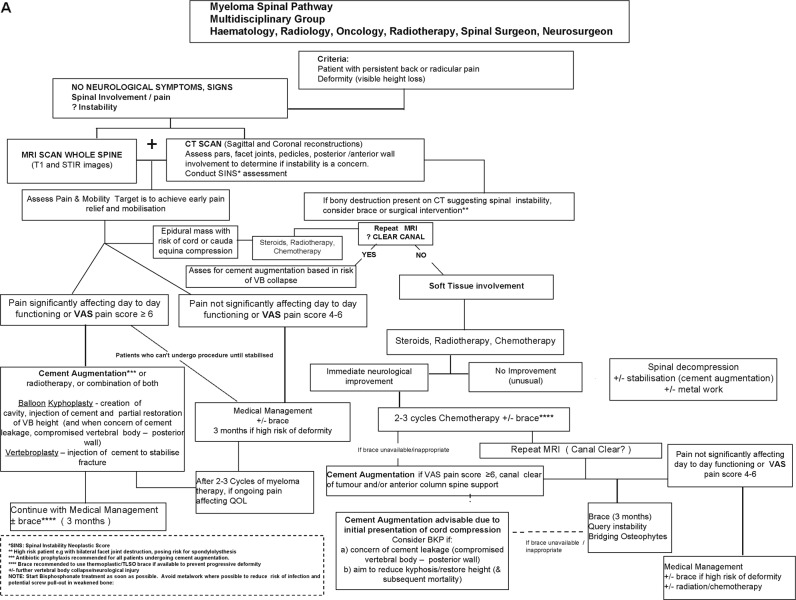

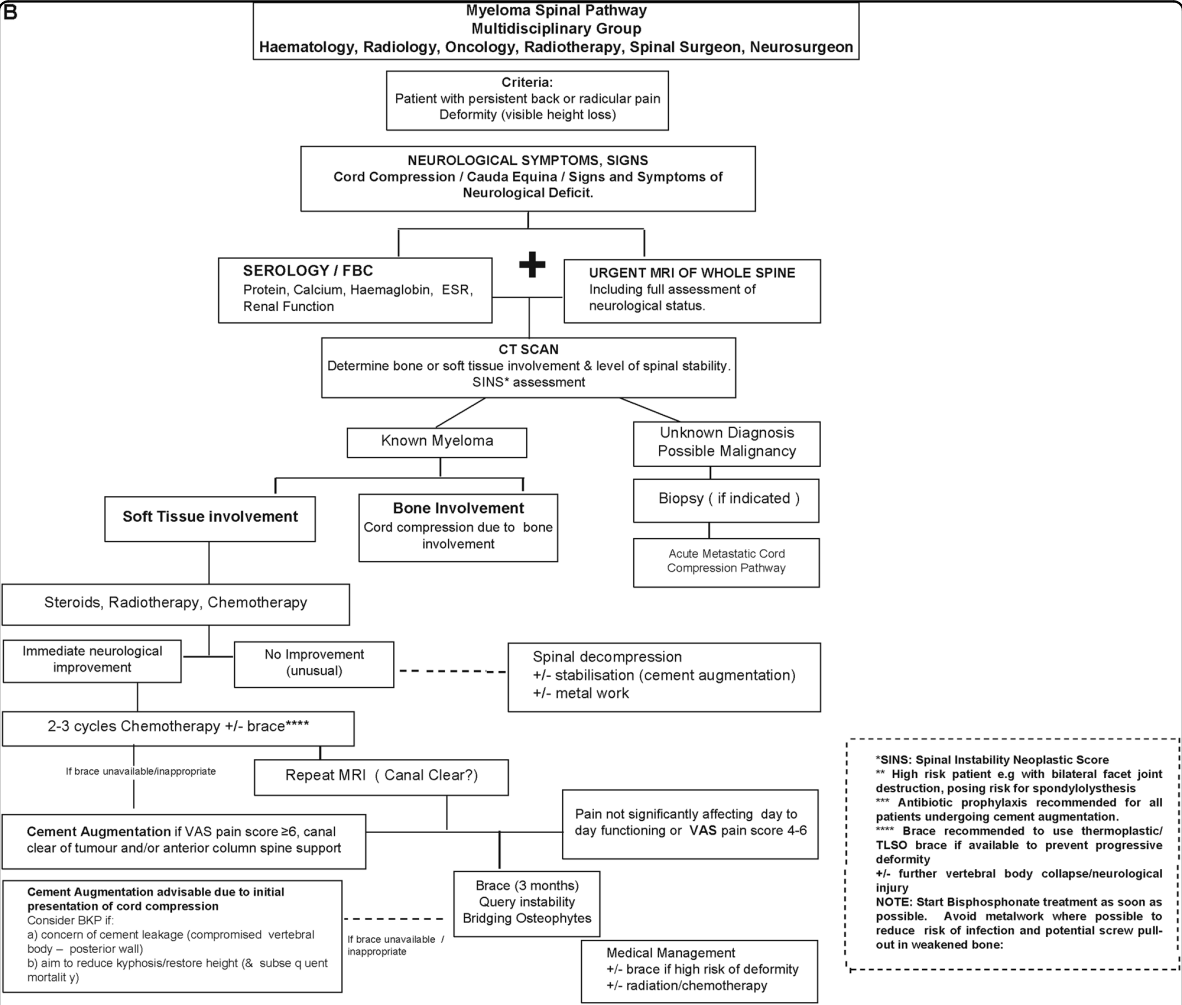
Myeloma Spinal Pathway. **a** Myeloma spinal pathway for myeloma patients presenting with spinal disease with no neurological symptoms. **b** Myeloma spinal pathway for myeloma patients presenting with spinal disease and associated neurological symptoms

The following is the consensus statement for recommendations for spinal support and cement augmentation from the International Myeloma Working Group. MM patients with significant pain at a fracture site should be offered a BKP or PV procedure and the procedure should be performed within 4–8 weeks unless there are medical contraindications (Tables 1 and 2, Fig. 1a, b).

**Table 1:** Indications for cement augmentation

**(1) Absolute indications for cement augmentation of a vertebral body or bodies due to fracture:**
• Persistent significant pain from a fractured vertebral body confirmed on MRI scanning with STIR images. This fracture could be acute, sub-acute or chronic (often has a fracture cleft) and has not healed
• Persistent significant symptoms which have not resolved with normal conservative measures after 4 weeks of treatment affecting daily activities
• Significant pain due to a fractured vertebral body affecting activity
• Significant pain associated with significant change in disability in conjunction with a new event
• Acute patient-delayed for medical reasons
• Selective chronic fractures
• Complications for myeloma should be treated first and pain is not defined by a specific VAS number
• Timing is important, especially newly diagnosed patients. Immediate referral for treatment for very severe pain requiring high dose of analgesics
**(2) Relative indications for cement augmentation of a vertebral body or bodies due to fracture:**
• Fracture of the thoracolumbar junction (T10–L2) that could result in a significant kyphotic deformity and therefore morbidity
• Loss of vertebral body height (progressive as evidenced by sequential erect x-rays)
• Posterior wall defect or destruction of a pedicle/pars which may potentially render the affected area of the spine unstable and at risk of fracture/neurological insult new tumour classification system to delineate vertebral bodies at risk of impending fracture as a result of metastatic spinal disease^82,83^. May be used for classification for myeloma patients as well but this needs to be myeloma spinal disease validated
**(3) Conditional or prophylactic indications for cement augmentation of a vertebral body or bodies due to fracture:**
**(A)** **Loss of vertebral height sufficient to affect functional activities**
• Fracture at T10–L2 (thoraco-lumbar junction) consider cement augmentation; below L2 is not as significant
• Only if progression over time; follow up with standard x-rays every 1–3 months
**(B)** **Risk of impending fracture**
• Need to take into consideration the aggressive nature of the disease and patient activity
• “Impending fractures” hard to determine
• Need for clinical trials

**Table 2:** Immediate vertebral cement augmentation

**Acute VCF with severe pain VAS** **≥** **6**
• However, often patients can be temporally stabilised in thermostatic TLSO (thoracolumbar sacro orthosis) to adequately control their pain while medical management is initiated
• Following 1–2 cycles of chemotherapy if patients present with poor performance status, septic, or have hyperviscosity problems that can be contraindications to undergo the procedure. Patients can be still treated with cement augmentation if still clinically indicated. The analgesics, bisphosphonate and chemotherapy treatment can provide pain relief and may alleviate some of the fracture pain.
**Subacute VAS 4–6**
• Patients with VCFs that are borderline should be treated with chemotherapy, bisphosphonates and conventional pain relief measures and if these fail then cement augmentation should be considered. If the pain persists or worsens or there is a risk for further vertebral collapse, then early intervention is required if stabilising the spinal structure and/or restoring the vertebral body height are critical.
• If the pain persists at the site of a previously diagnosed fracture the cement augmentation is still indicated if the pain is thought to be fracture and not facet joint related pain. These patients often have a fracture cleft in the vertebral body on the MRI imaging.
• VAS 1–3 Watchful surveillance with periodic skeletal survey (or other imaging as appropriate)

*VAS* visual analogue pain scores, *VCF* vertebral compression fractures

### Identification of patients suitable for vertebral augmentation

- Careful assessment to determine the severity and site of the pain^61^. Patients with acute fracture pain should be considered and not the patient with facet joint-related pain. The clinical picture should be confirmed with MRI scanning. The most useful MRI images to show an acute or on-going painful fracture are the sagittal STIR and T1 weighted sequences. The T1 weighted images may be more helpful in diagnosing a VCF in a MM patient than the STIR images. In addition, T1 images may also show the fracture line.
- MRI is crucial to document any radiological nerve root/cauda equina or spinal cord compression.
- CT scan with sagittal and coronal reconstructions may be needed to assess if there is spinal instability. A SINS classification can be helpful when determining the stability of the spine. If there is a posterior wall defect or pedicle/facet joint involvement, then CT can determine the safety of cement placement within the vertebral body. If patients get a recurrence of pain after a successful cement augmentation, then sagittal T1 and STIR images of the spine should be repeated to see if there is a new fracture that could develop following myeloma treatment.
- Assessment of myeloma disease status and therefore risk related to anaesthetic and any cement augmentation procedure. This includes potential anti-myeloma treatment requirements and risk for infection and bleeding. Coordination of procedure with treating haematologist/oncologist to avoid anaemia, leukopaenia and/or thrombocytopaenia related to systemic anti-myeloma therapy.

### Timing of vertebral cement augmentation (PV or BKP)

The CAFÉ Trial investigated early intervention in cancer patients who had VCF’s treated with BKP. The functional outcome (RDQ) was superior for the patients having BKP in the 1st month compared to the patients who received non-surgical treatment. The patients in the BKP group showed a marked reduction in back pain and required less pain relief. This is important for myeloma patients since most of them have a degree of renal impairment. In addition, improvement of function and mobility can reduce thrombotic and infection risk. Therefore, early intervention within 4–8 weeks^62,63^ of VCF’s with cement augmentation not only treats the pain associated with the fracture but in addition improves clinical outcome and QoL^52^ (Table 2).

### Number of levels to be considered for treatment

For patients with multiple VCF’s and significant pain, the maximum number of levels that should be augmented at a time should be determined by the operator. There is no upper limit for total number of vertebrae that should be treated in one intervention. The panel’s recommendation is that it is appropriate to treat up to 3 levels at a time and any decision treating more levels than this should be taken with caution. The reason for this is that cement embolus to the lungs may occur compromising respiratory function. Cement augmentation without cement leakage into the disc above or below should not increase the risk of adjacent vertebral body fracture. Cement leakage rates with BKP are reported to be less than with VP^28,31^. This is also the consensus of the panel.

### The highest level of cement augmentation

Cement augmentation of the spine is possible at all spinal levels. The C2 vertebral body can be augmented via a trans-oral or submandibular route. The C3–C7 vertebral bodies can be accessed and augmented through a standard open anterior cervical approach^64–66^ or percutaneously if the experience is available. The thoracic and lumbar vertebral bodies can be augmented with cement in the standard transpedicular or extrapedicular approach. Pain due to fractures from T1 to T4 rarely needs to be treated with cement augmentation because the pain usually settles with conservative management. Sarcroplasty can be performed if there is evidence of sacral insufficiency fractures.

### The method of vertebral cement augmentation

Published studies report contradicting results for cement augmentation^38–40,48,67–71^. Although there has been a change in emphasis from VP to BKP, the evidence for one procedure over the other is debatable. A meta-analysis of randomised and non-randomised trials of VP, BKP and NSM in patients with VCF due to osteoporosis^31^ has found that BKP was better than VP or NSM in reducing disability. Both BKP and VP were better for reducing pain (mostly during the first 8 weeks) and subsequent fracture risk (by about 50%) when compared to NSM. There was no difference between BKP and VP for these parameters. Cement leakage into the canal, lungs or other major organs was less for BKP than for VP. BKP was better in restoring mid-vertebral height and in changing kyphotic angle than VP and was also associated with less incidence of refracture. BKP, which involves inflation of a balloon tamp to create a void in the vertebral body, controls the delivery of cement better than PV. Patients with multiple VCF’s may become very kyphotic in the thoracic and lumbar regions of the spine. A hyperkyphosis results in a positive sagittal alignment also termed sagittal imbalance. Patients with a positive sagittal balance find it more difficult to stand in the upright posture and in attempting to do so expend more energy. Poor sagittal alignment has been shown to be a strong predictor of disability^72^. There has been debate as to the potential for BKP and PV to restore vertebral body height following a VCF. Some papers however report an improvement in the Cobb angle (degree of kyphosis) following BKP^50,54,73,74^ for VCF’s related to MM. Similar outcomes of KIVA implant to BKP vertebral augmentation were reported in patients with VCFs secondary to cancer and osteoporosis^75–79^. More research is needed to answer this question definitively. In addition, direct comparison of the complications of NSM, VP and BKP and the optimal timing for VCF treatment in MM patients are questions that could be answered in a prospective randomised, controlled clinical trial.

### Use of radiotherapy

The use of radiotherapy for local disease control and palliation should be used judiciously and sparingly depending on the patient’s presentation, need for urgent response, and prior treatment history and response. MRI and CT scans are crucial to differentiate between a soft tissue myelomatous mass in the spinal canal from bony encroachment. The reason for this is that radiation therapy is very effective in reducing the size of a soft tissue myelomatous mass but not effective if there is bony neural compression and does not stabilise the VCF.

Radiotherapy should be limited as much as possible to spare the patient’s marrow function. Current systemic combination therapies of steroids with novel agents work rapidly and should decrease the need for palliative radiotherapy. Radiation therapy may be appropriate for:

1. Patients with a soft tissue mass or plasmacytoma that has not resolved with systemic therapy
2. Patients who cannot receive systemic therapy
3. Relapsed refractory patients
4. Palliative approach for poor performance status patients
5. When mass is associated with severe pain
6. Location of plasmacytoma precluding use of BKP or PV; e.g. tumour impacting posterior part of the vertebral body close to spinal cord and nerves.

**Fig. 2:**
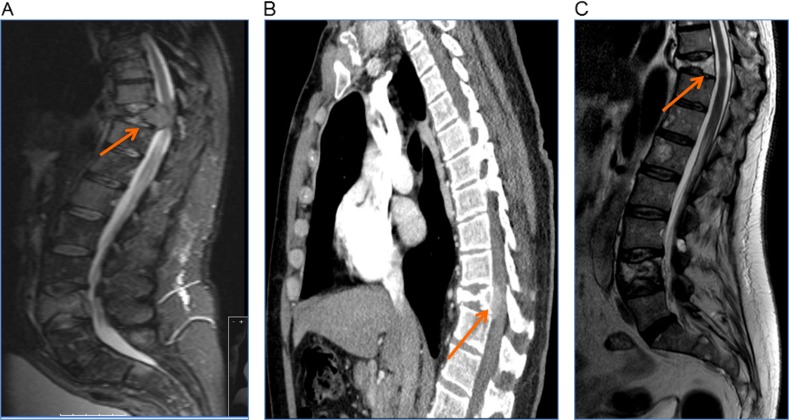
A 57-year-old male presented with bilateral leg weakness (3/5 MRC), sensory disturbances and back pain, catheterised with good anal tone. **a** Initial MRI revealed T10 collapse with tumour in canal causing spinal cord compression. **b** Soft tissue CT windowing confirmed that it was soft tissue tumour without bone element in the spinal canal. The patient was treated with dexamethasone and radiotherapy for cord compression, had TLSO brace fitted for relative stability and received 2 cycles of chemotherapy for kappa light chain myeloma. **c** MRI was repeated for persistent severe back pain (VAS 8/10) and reassessment of cord compression. Clinically power was 5/5 in both legs. The MRI confirmed soft tissue mass response and spinal stability. Patient had cement augmentation with BKP at T10 to relieve the pain and 24 h later VAS was 1/10

Receiving radiotherapy and the dose of previous radiotherapy are not contraindications for cement augmentation (PV or BKP) if it is needed. The need and timing of a cement augmentation procedure for patients that have been irradiated depends on the patient’s pain (Fig. 2). The cement augmentation procedures are performed through small stab incisions and therefore the usual concerns over wound healing do not exist. Patients that have a posterior wall defect (associated with a soft tissue mass encroaching the spinal canal) or pedicle/facet joint involvement may need supplementary cement augmentation despite having received radiotherapy to stabilise the anterior and middle columns of the spine. Planned vertebral augmentation 4–8 weeks later (or after the second cycle of chemotherapy) is appropriate for patients with relative vertebral instability. The aim of the cement augmentation is to halt further collapse of the fractured vertebral body that could result in progressive kyphosis and secondary neural compromise.

Overall, the proposed algorithm for spinal support in myeloma presenting with VCFs or spinal cord compression is summarised in a flow diagram in Fig. 1b. The first major decision point is the presence or absence of signs and/or symptoms of neurological deficit. Obviously, if there is, this is an urgent matter and recommendations proceed accordingly. Once the situation has been assessed, stabilised and treated, then cement augmentation can be an option if there is persistent pain. For patients without neurologic compromise, imaging and multi-disciplinary assessment are recommended as the basis for consideration of cement augmentation. Integration within the total treatment schema is the primary plan.

## Discussion

The prognosis of patients with myeloma has improved considerably over the last 15 years because of the advances in Haematological Oncology. We therefore need to become more proficient at treating the associated medical/surgical complications related to the disease. One such complication is one or more VCF’s of the spine. Patients may present with significant spinal fracture pain or neurological compromise. These signs and symptoms may present at the time of the index procedure or when there is a relapse of the disease. Essentially, myeloma patients that present with spinal symptoms and signs need to be assessed to establish the source and nature of their pain and presence/absence of neurological compromise and spinal instability.

Those that present with neurological compromise may have spinal cord, cauda equina or nerve root compression. It is imperative, in patients with neurological compromise, to get not only an MRI scan with STIR and T1 weighted images but also a CT scan (with soft tissue windowing) to delineate whether it is bone or soft tissue compromising the neurological structures. Soft tissue in the spinal canal due to a myelomatous deposit is usually very sensitive to treatment with chemotherapy/radiotherapy/steroids and therefore the neural compression can be treated by these modalities without the need for surgical decompression. Ideally, one would like to avoid any instrumentation in myeloma patients if possible because of the risk of subsequent metalwork/deep spinal infection during periods of immunocompromise. However, if there is significant spinal cord compromise/cauda equina compression then surgical decompression may be needed as an emergency and an immediate spinal surgical consultation should be sought on all such patients. Once the spinal cord/cauda equine compression has been treated with steroids/chemotherapy/radiotherapy, cement augmentation may be needed to alleviate the pain associated with the fracture or to restore spinal stability. It is uncommon for a myeloma patient to present with neurological compromise because of bony encroachment but if it occurs, radiotherapy/chemotherapy/steroids will not be an effective treatment. Surgery may be needed in this cohort of patients.

Patients who present with spinal pain, but no neurological compromise should have an MRI scan performed with STIR and T1 weighted images to detect any spinal fractures. The T1 weighted images may be better than the STIR images in highlighting the fracture line in vertebrae infiltrated with a myelomatous deposit. If one has concerns about the spinal stability because of a posterior wall defect or pedicle/facet joint/pars involvement, then a CT scan with sagittal and coronal reconstructions can be very helpful. Patients can have quite significant bony defects but still be structurally stable in an orthotic brace^80^. A brace may be all that is needed in patients with a spinal fracture if they can mobilise without significant pain. The external orthosis will also keep the patients in the upright posture (and potentially prevent the development of a kyphotic deformity) while their fractures heal. Patients who present with spinal pain and have a new diagnosis of myeloma may need, depending on systemic symptoms, to have their disease controlled with chemotherapy prior to any consideration for cement augmentation. The chemotherapy immune-compromises the patients and therefore the correct timing of cement augmentation should be a multi-disciplinary decision. Antibiotic prophylaxis in the peri-operative period is strongly advised to avoid infection. The orthotic brace can be a very useful tool to control the pain to an acceptable level while the disease is being treated with the first couple of cycles of chemotherapy.

Another important aspect of the treatment in myeloma patients involves bisphosphonate therapy. This drug treatment clearly helps to stabilise the bone density in patients with myeloma but, in addition may have a positive effect in producing an external scaffold of bone around the vertebral bodies to confer them extra stability. This external scaffold, which has been described as DISH in prior publications^81^ in patients with myeloma, may decrease the need for spinal fixation in patients otherwise thought to be at risk of spinal instability because of involvement of all three bony spinal columns^57^.

## Conclusion

The prognosis of MM is continually improving due to medical advances. The treatment of myeloma with chemo- immunotherapeutic agents and autologous stem cell transplantation renders the patient immunocompromised for periods of time, exposing them to infection. Spinal fixation has been employed traditionally to treat myeloma patients when decompression and stabilisation were deemed to be essential. However, it is well established that in situ instrumentation is at risk of getting infected when the patients are in an immunocompromised state. If the metalwork gets infected, then the consequences can be catastrophic. Cement augmentation is a very effective way of stabilising the anterior and middle spinal columns without the need for metalwork fixation. It is an excellent way to relieve the pain from a VCF. The myeloma spine treated with bisphosphonates appears to produce an external scaffold of bone that stabilises even the most moth-eaten spinal elements once the disease process is under control. An external orthosis can be very effective when trying to achieve pain relief from a fracture. It also helps to maintain the correct sagittal balance in patients with multiple fractures while they heal or before they are treated with cement augmentation. The development of radiofrequency ablation in combination with cement augmentation procedures is currently under investigation with encouraging results.
